# Lionfish envenomation on the Brazilian coast: first report

**DOI:** 10.1590/0037-8682-0241-2022

**Published:** 2022-10-24

**Authors:** Vidal Haddad, Tommaso Giarrizzo, Marcelo de Oliveira Soares

**Affiliations:** 1 Universidade Estadual Paulista, Faculdade de Medicina de Botucatu, Botucatu, SP, Brasil.; 2 Universidade Federal do Ceará, Instituto de Ciências do Mar (Labomar), Fortaleza, CE, Brasil.; 3 Leibniz Center for Tropical Marine Research (ZMT), Bremen, Germany.

**FIGURE 1:**
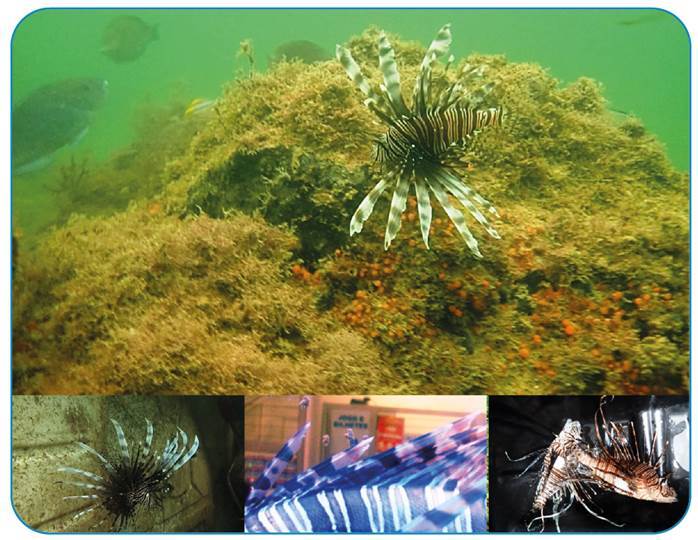
Lionfish (*Pterois* spp.) and venomous apparatus in the rays of the fins on the Ceará coast (Brazil). **Photos:** Tommaso Giarrizzo.

The lionfish is a fish of the Scorpaenidae family (to which scorpionfish, mangangás, and beatriz belong). They are venomous fish carrying neuromuscular toxins with systemic effects on the rays of their fins (mainly dorsal), which can have serious consequences for victims^1^ (Figure 1). The lionfish, originally from the Indo-Pacific, is considered to be one of the most threatening, invasive species in the Atlantic Ocean. 

Over the past 30 years, this species has become established eastward off the coast of North Carolina (US), across the Caribbean Sea, Gulf of Mexico, and down to Brazilian waters (in the states of Pará and Amapá, and the archipelago of Fernando de Noronha). On the Brazilian semi-arid coast, it has already been recorded in shallow-water habitats (1-9 m depth) on the Piauí state and the west coast of Ceará state. The most common species in the invasion of the Atlantic Ocean is *Pterois volitans*, and the potential for envenomation in humans is high because of the beauty of the fish and the curiosity aroused by a fish previously not present on the Brazilian coast, in addition to the risks caused by stepping on the fish in shallow water and/or by incorrect handling. 

This report describes the first recorded envenomation in the country using accurate data. A male fisherman, 24 years old, pierced his foot in seven locations (four on the dorsum and three on the side of the foot) on the fin rays of a lionfish while working in a large tidal weir, which is a fixed artisanal fishing trap composed of two fences that guide tidally migrating fish into a catch chamber and is cleaned at low tide. The injury occurred on the beach of Bitupitá, in the municipality of Barroquinha, in Ceará state. Shortly after the perforation, he had edema and erythema at the site, excruciating pain, fever, and reported seizures and cardiac arrest (seizures and cardiac arrest were later ruled out; however, the wound site developed ecchymoses at the inoculation points). Health services were performed in a timely manner (1 h 30 min), where anti-inflammatory drugs, analgesics, and wound dressings were administered. The patient was discharged with a recommendation to return, if necessary. As more specimens of lionfish were caught in the same place, there was no doubt about the cause of the envenomation, and the patient's clinical progress after hospital treatment was satisfactory. 

**FIGURE 2:**
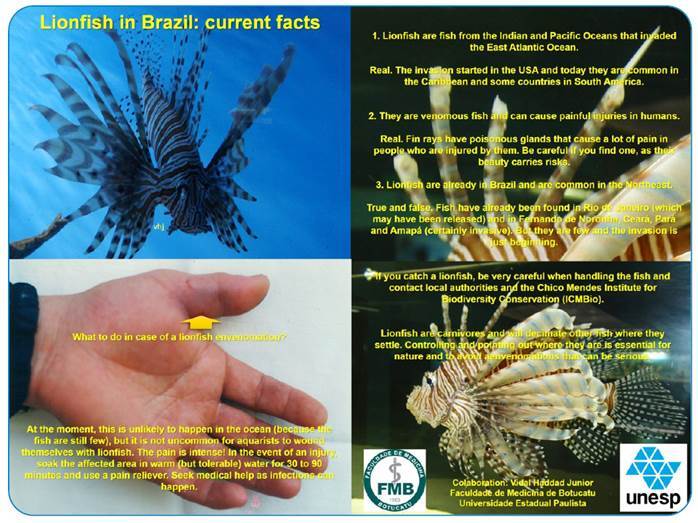
Flyer with information regarding the lionfish and the initial treatment for possible envenomation in the Brazilian Coast. Vidal Haddad Junior.

Envenomation caused by fish of the Scorpaenidae family can be serious, depending on the amount of venom inoculated. Multiple perforations may precipitate seizures and cardiac arrest since toxins have a systemic neuromuscular effect. Although lionfish usually do not cause serious disorders, multiple inoculations may be associated with increased severity of cases^2^. With the increase in the capture of these fish on the Brazilian coast, this case should serve as a warning to the population and medical teams (Figure 2).
